# Prevalence and factors associated with external HIV-related stigma in the South African population: Results from the 2017 population-based household survey

**DOI:** 10.1371/journal.pone.0309694

**Published:** 2024-09-03

**Authors:** Vuyelwa Mehlomakulu, Musawenkosi Mabaso, Sean Jooste, Allanise Cloete, Sizulu Moyo, Leickness Simbayi

**Affiliations:** Public Health, Societies and Belonging Division, Human Sciences Research Council, Pretoria, South Africa; International AIDS Vaccine Initiative, UNITED STATES OF AMERICA

## Abstract

External HIV-related stigma remains pervasive, and its effect debilitating among PLHIV in South Africa, even though the country has made many advances against HIV. External HIV-related stigma impedes both HIV prevention and access to health care and reduces the quality of treatment and care received. This study examined the prevalence of and factors associated with higher levels of HIV-related stigma among youth and adults 15 years and older in South Africa. The analysis used a nationally representative population-based household survey data collected using a multistage cluster random sampling design. Exploratory factor analysis was used to calculate the primary outcome (higher and lower HIV stigma index scores above and below the mean, respectively), based on the total number of factors retained from the 10 item self-reported questions relating to attitudes and beliefs against PLHIV. Bivariate and multivariate generalised linear models with a log link and binomial distribution were fitted to estimate crude and adjusted risk ratios (ARR) with 95% confidence intervals (CI) for factors associated with external HIV-related stigma. Of 38 919 respondents, 49% (49.8%; 95% CI: 48.6–51.1) were categorised as having higher levels of external HIV-related stigma. Higher levels of HIV-related stigma were significantly associated with those who had secondary level education than those with no education/primary education [ARR = 1.14 (95% CI: 1.05–1.24), p = 0.002], those employed than unemployed [ARR = 1.08 (95% CI: 1.02–1.14), p = 0.006], those in rural areas than urban areas [ARR = 1.15 (95% CI: 1.07–1.23), p<0.001], those who were aware of their HIV status than not aware [ARR = 1.34 (95% CI: 1.12–1.61), p<0.001], those who were HIV positive than HIV negative [ARR = 1.09 (95% CI: 1.02–1.17), p = 0.018], and those with no correct HIV knowledge and myth rejection than their counterparts [ARR = 1.09 (95% CI: 1.03–1.15), p = 0.002]. The findings highlight the need for peer-facilitated HIV-stigma reduction interventions targeting all types of educational institutions and the strengthening of work-based interventions. The findings emphasise the prioritisation of rural informal settings/tribal areas when developing and implementing HIV stigma reduction interventions. The study suggests that stigma reduction should be considered an important component of HIV testing and awareness. Addressing public misconceptions about HIV can mitigate externalised stigma.

## Introduction

HIV-related stigma is one of the biggest challenges facing people living with HIV (PLHIV) globally [1]. External HIV-related stigma is received and/or an enacted form of this social phenomenon that is associated with public blame for contracting HIV and refers to negative beliefs, feelings, and attitudes towards PLHIV [2, 3] Several studies have shown that a high proportion of PLHIV are stigmatised by the community members they share their lives with [2, 4, 5].

Evidence shows that despite continued widespread information, education, and communication campaigns to raise awareness about HIV, external HIV-related stigma is still highly prevalent in sub-Saharan Africa. A multicountry cross-sectional study using data from the Demographic and Health Surveys of 12 sub-Saharan African countries found that the prevalence of negative attitudes toward PLHIV was 36.2% [6]. The 2012 South African national HIV household survey reported that 38% of PLHIV portrayed HIV-related stigma attitudes towards PLHIV [7], and the national stigma index study in South Africa revealed that 36% of PLHIV experienced external HIV-related stigma [8].

External HIV-related stigma hinders accessibility to HIV-related services and support programmes [2, 3]. This has been shown to negatively affect adherence to antiretrovirals and viral load suppression resulting in poor health outcomes [9, 10]. External HIV-related stigma has also been associated with delaying diagnosis and non-disclosure among PLHIV and, therefore, reducing linkage to care and delaying achievement of the UNAIDS 95-95-95 goals [11].

External HIV-related stigma also impacts HIV prevention as PLHIV continue to engage in risky sexual practices such as having unprotected sex because discussions about the need for safer sex often lead to questions about a partner’s serostatus [12]. Similarly, fear of rejection and intimate partner violence may lead some to hide their positive HIV status from sexual partners [12]. Risk reduction, resilience, and coping interventions have been instrumental in reducing the negative impact of external HIV-related stigma on the lives of PLHIV [13]. However, many factors associated with external HIV-related stigma may also impact the effectiveness of stigma-reducing interventions [14]. Therefore, identifying factors associated with HIV-related stigma might help redesign or reinforce these interventions.

Externalised HIV-related stigma is underpinned by many factors, including socio-demographic factors such as race, sex, marital status, level of education, socioeconomic status, and place of residence [2, 6, 15]. Factors associated with externalised stigma also include poor HIV knowledge and misconceptions about HIV [16, 17]. The literature also suggests that there is a link between HIV-related knowledge, HIV serostatus awareness/disclosure, and stigma due to psychological and social stresses associated with a positive HIV test [16–21]. However, few studies have examined the drivers of HIV-related stigma at a population level.

South Africa has the largest number of PLHIV globally, and external HIV-related persists after more than 20 years of the fight against HIV despite efforts to address it [22, 23]. An improved understanding of the levels of external HIV-related stigma and associated factors at the population level is important for developing innovative and targeted interventions at a national level. Therefore, this paper examines the prevalence of external HIV-related stigma and associated factors in the general population in South Africa.

## Methods

### Ethical considerations

The original survey was approved by the Human Sciences Research Council ethics board under Ethics Clearance of HSRC Research Ethics Committee Protocol No REC4/18/11/15.

### Data source and study sample

This study used data from the 2017 South African National HIV Prevalence, Incidence and Behaviour Survey, a nationally representative population-based household survey collected using a multistage cluster random sampling design described in detail elsewhere [22]. Data collection started on the 4th of December 2016 and ended on the 31st of January 2018. The data were collected using a complex multistage-stratified cluster sampling design. Using the 2015 national population sampling frame of 84 907 small area layers (SALs) developed by Statistics South Africa [24], 1000 SAL were randomly sampled across the country. A systematic random sample of 15 households was sampled within each SAL. All individuals living in a selected household were eligible for the survey.

Detailed questionnaires for the household and age-appropriate individual behavioural questionnaires were administered to all consenting eligible adults aged 18 years and older and all assenting eligible children aged 12–17 years old who also had parental or guardian consent. Parents or guardians completed the questionnaires on behalf of eligible children under the age of 12 years. Interview data was collected digitally using tablet computers with Census Survey Processing (CS Pro) software (US Census Bureau). In addition, dried blood spot (DBS) specimens were collected from consenting participants to determine, among other tests, HIV serostatus [22]. The sub-sample used in this study comprised youth and adults aged 15 years and older who responded to questions on HIV-related stigma.

### Measures

#### Dependent variables

The primary outcome measure of the HIV stigma scale was based on a question relating to attitudes and beliefs against PLHIV with 10 items listed below:

1. If you knew that a shopkeeper or food seller had HIV, would you buy food from them?
2. Would you buy fresh vegetables from a shopkeeper or vendor if you knew that this person had HIV?
3. Would you be willing to care for a family member with AIDS?
4. If a teacher has HIV but is not sick, should he or she be allowed to continue teaching?
5. Is it a waste of money to train or give a promotion to someone with HIV/AIDS?
6. Would you want to keep the HIV-positive status of a family member a secret?
7. Are you comfortable talking to at least one member of your family about HIV/AIDS?
8. A person would be foolish to marry a person who is living with HIV/AIDS?
9. If a pupil has HIV but not sick, should he or she be allowed to continue to go to school?
10. Do you think children living with HIV should be able to attend school with children who are HIV-negative?

The respondents had to answer “Yes”, “No” and “Don’t know”.

Responses were assessed using exploratory factor analysis to determine the factor structure and items to be retained. Cronbach’s Alpha was used to assess the reliability of individual items used in the analysis. A varimax orthogonal rotation assessed the underlying domains and reduced the number of items retained [25]. Eigenvalues were used to identify factors that account for most variance within the items. Variables with a factor loading of at least 0.4 and factors with an eigenvalue of at least one retained for item analysis [26]. HIV stigma index scores were constructed by adding the total number of factors of retained items. The index score was then calculated using the mean as the cut-off point to categorise scores into higher or lower [27]. This approach has been used in different contexts in several countries [28–32]. The respondents whose scores were above the mean were classified as having higher levels of HIV stigma, and respondents with scores below the mean were classified as having lower levels of HIV stigma. The dichotomised HIV index score was coded into a binary outcome measure of external HIV-related stigma (0 = lower levels of stigma and 1 = higher levels of stigma). Cronbach’s alpha coefficient for items used to measure higher external HIV-related stigma was 0.8.

#### Independent variables

Explanatory variables included demographic factors were age groups in years (15–24, 25–49, 50 and above), sex (Male, Female), race groups (Black African, Other), marital status (Married, Never Married), educational level (No education/Primary, Secondary, Tertiary), employment status (Employed, Not employed), and locality type (Urban, Rural informal/Tribal areas, Rural/Farms). We also included HIV-related variables such as ever tested for HIV (Yes = tested for HIV, No = Never tested for HIV), awareness of HIV status (Yes = aware of HIV status, No = Not aware of HIV status), HIV status (Negative, Positive based in testing of survey sample), self-perception of the risk of HIV Infection (Low, High), and correct HIV knowledge and rejection of myths (Yes = correct knowledge, No = incorrect knowledge) based on the following questions: “AIDS can be cured; a person reduce the risk of HIV by having fewer sexual partners; a healthy-looking person can have HIV; a person gets HIV by sharing food with someone who is infected; a person reduce the risk of getting HIV by using a condom every time he/she has sex?”

#### Data analysis

Data were weighted and benchmarked to the 2017 mid-year population in South Africa. All analyses were conducted using STATA 15.0 software (Stata Corporation, College Station, TX, United States). Descriptive statistics (frequencies and percentages) were used to summarise study characteristics and external HIV-related stigma. Pearson’s Chi-squared test assessed differences between categorical variables. Bivariate and multivariate generalised linear models with a log link and binomial distribution were used to estimate risk ratios (RR) for factors associated with high levels of external HIV-related stigma. All factors significantly associated with externalised HIV-related stigma in bivariate models (p-value less than 0.2) were entered into a multivariate model to evaluate the independent effect of these variables. In the final model, adjusted risk ratios (ARR) with 95% confidence intervals (CI) and a p-value < 0.05 were considered statistically significant.

## Results

### Sample characteristics

The study sample consisted of 38 919 participants (Table 1). Over half were aged 25–49 years (53.4%) and were males (51.7%). Most were Black African (79.2%), had completed secondary school education (66.8%), never married (68.0%), were unemployed (64.0%), and resided in urban areas (66.3%). The majority reported that they had tested for HIV (74%), were aware of HIV status (96.6%), were HIV negative (80.7%), had a low self-perceived risk of contracting HIV (83.4%), and had low correct HIV knowledge and myth rejection (63.7%).

**Table 1:** Characteristics of the study sample aged 15 years and older (n = 38 919), South Africa 2017.

Variables	Total	%
**Age groups (years)**		
15 to 24	10795	24.1
25 to 49	17936	53.4
50 years and older	10188	22.4
**Sex**		
Male	15967	48.3
Female	22952	51.7
**Race groups**		
Black African	30445	79.2
Other	8474	20.8
**Marital status**		
Married	10424	32.0
Never married	24775	68.0
**Education level**		
No education/Primary	5781	17.0
Secondary	18521	66.8
Tertiary	3560	16.2
**Employment status**		
No	26608	64.0
Yes	11831	36.0
**Locality type**		
Urban	21213	66.3
Rural informal (tribal areas)	13504	28.2
Rural (farms)	4202	5.5
**Ever tested for HIV**		
No	11229	26.0
Yes	27508	74.0
**Awareness of HIV status**		
No	969	3.4
Yes	26530	96.6
**HIV status**		
Negative	21185	80.7
Positive	5364	19.3
**Self-perceived risk of HIV**		
Low	29979	83.4
High	5104	16.6
**Correct HIV knowledge and myth rejection**		
No	24883	63.7
Yes	13994	36.3

Subtotals do not all equal to the total due to non-response and/or missing data; CI, confidence intervals.

### Prevalence of external HIV-related stigma

Of 38 919 respondents, 49% (49.8%; 95% CI: 48.6–51.1) participants were categorised as having higher levels of external HIV-related stigma for our analysis. Table 2 shows higher levels of external HIV-related stigma by sample characteristics. The prevalence of higher levels of external HIV-related stigma were found among those aged 25–49 years, females, Black Africans, those who never married, those with secondary education, the employed, and those residing in rural informal/tribal areas. The prevalence of higher levels of external HIV-related stigma was also found among those who ever tested for HIV, those who were aware of their status, those who tested positive for HIV, those who perceived themselves as at high risk of HIV infection, and those with no correct HIV knowledge and myth rejection.

**Table 2:** Prevalence of higher levels of external HIV-related stigma by socio-demographic characteristics and HIV-related factors, South Africa 2017.

Variables	n	%	95% CI	p-value
**Age groups (years)**				<0.001
15 to 24	10795	23.7	22.7–24.8	
25 to 49	17936	57.9	56.6–59.0	
50 and older	10188	18.4	17.4–19.5	
**Sex**				0.015
Male	15967	47.4	46.2–48.6	
Female	22952	52.6	51.4–53.8	
**Race**				<0.001
Black African	30445	84.6	82.5–86.5	
Other	8474	15.4	13.5–17.5	
**Marital status**				<0.001
Married	10424	30.5	28.6–32.4	
Never married	24775	69.5	67.6–71.4	
**Education level**				<0.001
No education/Primary	5781	14.2	13.2–15.3	
Secondary	18521	69.3	67.5–71.0	
Tertiary	3560	16.4	14.7–18.4	
**Employment status**				<0.001
No	26608	62.4	60.6–64.1	
Yes	11831	37.6	35.9–39.4	
**Locality type**				0.004
Urban	21213	64.7	60.9–68.3	
Rural informal/tribal areas	13504	30.1	26.5–33.8	
Rural/farm areas	4202	5.3	4.2–6.7	
**Ever tested for HIV**				<0.001
No	11229	22.4	21.1–23.7	
Yes	27508	77.6	76.3–78.9	
**Awareness of HIV status**				<0.001
Not aware	969	2.8	2.4–3.3	
Aware	26530	97.2	96.7–97.6	
**Survey HIV status**				<0.001
Negative	21185	77.5	76.1–78.8	
Positive	5364	22.5	21.2–23.9	
**Self-perceived risk of HIV**				0.015
Low	29979	82.5	81.3–83.7	
High	5104	17.5	16.3–18.7	
**Correct HIV knowledge and myth rejection**				<0.001
No	24883	59.8	58.2–61.4	
Yes	13994	40.2	38.6–41.8	

Subtotals do not all equal to the total due to non-response and/or missing data; CI, confidence intervals.

### Factors associated with external HIV-related stigma

#### Bivariate logistic regression models

Table 3 shows that higher levels of HIV-related stigma towards PLHIV were significantly more likely among those aged 24–49 years, females, Black Africans, those who never married, those with secondary and tertiary education levels, the employed, and those residing in rural informal/tribal areas. Higher levels of HIV-related stigma were significantly less likely among those who ever tested for HIV, those aware of HIV status, those who perceived themselves as at high risk of HIV infection, and those with correct HIV knowledge and myth rejection.

**Table 3:** Bivariate models of factors associated with higher levels of HIV-related stigma towards people living with HIV (PLHIV).

Variables	Crude RR	95% CI		p-value
**Age group (years)**				
15–24	ref.			
25–49	1.10	1.06	1.14	<0.001
50 and older	0.83	0.80	0.87	<0.001
**Sex**				
Male	ref.			
Female	0.83	0.80	0.87	<0.001
**Race**				
Black African	ref.			
Other	0.69	0.65	0.74	<0.001
**Marital status**				
Married	ref.			
Never married	1.07	1.03	1.12	0.001
**Education level**				
No education/Primary	ref.			
Secondary	1.24	1.18	1.30	<0.001
Tertiary	1.21	1.13	1.30	<0.001
**Employment status**				
Not employed	ref.			
Employed	1.07	1.03	1.11	<0.001
**Locality type**				
Urban	ref.			
Rural informal (tribal areas)	1.09	1.03	1.16	0.003
Rural (farms)	0.98	0.89	1.07	0.635
**Ever tested for HIV**				
No	ref.			
Yes	1.22	1.16	1.28	<0.001
**Awareness of HIV status**				
No	ref.			
Yes	1.22	1.08	1.36	0.001
**Survey HIV status**				
Negative	ref.			
Positive	1.21	1.16	1.27	<0.001
**Self-perceived risk of HIV**				
Low	ref.			
High	1.06	1.01	1.11	0.013
**Correct HIV knowledge and myth rejection**				
No	ref.			
Yes	1.18	1.14	1.22	<0.001

RR-risk ratios, CI-confidence intervals

#### Multivariate logistic regression model

Table 4 shows the final multivariate mode with adjusted risk ratios (ARR) for factors associated with higher levels of HIV-related stigma. Higher levels of HIV-related stigma were significantly associated with those who had secondary level education than those who had no education/primary education [ARR = 1.14 (95% CI: 1.05–1.24), p = 0.002], those who were employed than the unemployed [ARR = 1.08 (95% CI: 1.02–1.14), p = 0.006], those who resided in rural informal/tribal areas than those in urban areas [ARR = 1.15 (95% CI: 1.07–1.23), p<0.001]. Furthermore, higher levels of HIV-related stigma were significantly associated with who were not aware of their HIV status than those who were aware [ARR = 1.34 (95% CI: 1.12–1.61), p<0.00], those who were HIV positive than those who were HIV negative [ARR = 1.09 (95% CI: 1.02–1.17), p = 0.018] and with no correct HIV knowledge and myth rejection than their counterparts [ARR = 1.09 (95% CI: 1.03–1.15), p = 0.002].

**Table 4:** Final multivariate generalised linear model of factors associated with higher levels external HIV-related stigma towards people living with HIV (PLHIV).

Variables	ARR	95% CI		p-value
**Age group (years)**				
15–24	ref.			
25–49	1.00	0.93	1.07	0.935
50+	0.91	0.83	1.01	0.082
**Sex**				
Male	ref			
Female	1.05	0.99	1.10	0.093
**Marital status**				
Married	ref.			
Never married	1.03	0.97	1.10	0.364
**Education level**				
No education/Primary	ref.			
Secondary	1.14	1.05	1.24	0.002
Tertiary	1.06	0.95	1.19	0.276
**Employment status**				
Not employed	ref.			
Employed	1.08	1.02	1.14	0.006
**Locality type**				
Urban	ref.			
Rural informal (tribal areas)	1.15	1.07	1.23	<0.001
Rural (farms)	0.98	0.86	1.11	0.723
**Ever tested for HIV**				
No	ref.			
Yes	1.22	1.16	1.28	<0.001
Awareness of HIV status				
No	ref.			
Yes	1.09	1.02	1.17	0.018
Survey HIV status				
Negative	ref.			
Positive	1.21	1.16	1.27	<0.001
**Self-perceived risk of HIV**				
Low	ref.			
High	1.04	0.97	1.11	0.299
**Correct HIV knowledge and myth rejection**				
No	ref.			
Yes	1.09	1.03	1.15	0.002

ARR- adjusted risk ratios, CI-confidence intervals

## Discussion

This nationally representative study examined the prevalence and factors associated with higher levels of HIV-related stigma among youth and adults 15 years and older. The results revealed that 50% of respondents were categorised as having had higher levels of external HIV-related stigma. The 2014 South African study on external HIV-related stigma reported a 38% prevalence among the adult population [7]. Similarly, in Ethiopia, the 2016 Demographic and Health Survey found a 36% prevalence of high levels of external HIV-related stigma [2]. Furthermore, a study conducted in 15 sub-Saharan African countries reported an overall prevalence of 47%, with the highest external HIV-related stigma of 79.8% reported in Malawi [33]. These observations show that in sub-Saharan Africa, external HIV-related stigma remains relatively high, and this has been attributed to a number of social, cultural and structural factors [33, 34].

In the current study, the prevalence of higher external HIV-related stigma varied by socio-demographic and HIV-related factors, as expected. The final model showed that those with secondary education were more likely to report higher levels of external HIV-related stigma than those with no education/primary education. On the contrary, others found that stigmatising attitudes were more likely among those with low levels of educational attainment [2, 6, 34]. In agreement with current findings, others showed that higher levels of HIV-related stigma were associated with post-primary education [35, 36]. While educational attainment may be an important source of information and knowledge, school may also be the most important social sphere related to HIV stigma, where youth experience HIV stigma related to their status [35]. This suggests a need for peer-facilitated HIV stigma reduction intervention targeting all types of educational institutions, including schools.

The model showed that being employed was associated with higher external HIV-related stigma. Others found that those who were unemployed reported more stigmatising attitudes towards PLHIV [36, 37], and this has been attributed to employment wellness programmes which provide HIV-related information towards reducing stigmatising attitudes in the workplace [37]. However, PLHIV Stigma Index data records on work-related stigma indicate that there has been little improvement since 2000 and that HIV-related stigma enacted by co-workers remains pervasive [38]. There is a need to continue to review and modify HIV programmes and interventions aimed at reducing work-based HIV-related stigma towards delivering supportive workplaces for PLHIV [38].

The final model also showed that higher levels of external HIV-related stigma were associated with residing in rural informal/tribal areas, and these findings are consistent with other studies [39, 40]. Evidence shows that HIV-related stigma in rural and tribal communities is influenced by sociocultural norms and power structures that associate HIV with immoral sexual behaviour [41, 42]. These observations underscore the need to consider sociocultural and structural factors in developing and implementing stigma-reducing interventions, especially in rural settings [41].

Furthermore, the model showed that higher levels of external HIV-related stigma were associated with awareness of HIV status. However, other studies found that those who tested and were, therefore, aware of their HIV status were less likely to endorse stigmatising attitudes towards PLHIV [37, 40]. In addition, the model showed that higher levels of external HIV-related stigma were associated with HIV-positive status. This suggests that external HIV-related stigma does not only emanate from the general population but may also arise from PLHIV towards not only themselves but their peers as well. This can be attributed to internalised or self-stigma wherein the external stigma becomes internalised [43], suggesting even stronger feelings about their HIV status or their peers who are living with HIV. A similar finding is reported in the study done in Morocco, wherein those who experienced external HIV-related stigma presented with higher internal stigma scores, alluding to an association between these two phenomena [44]. These observations emphasise the importance of empowering PLHIV to take the lead in advocacy activities against stigma.

### Limitations

The items used to construct the external HIV-related stigma were based on self-report and may be affected by social desirability bias. The cross-sectional design makes it impossible to infer causation in any of the associations reported in this study. The measure of HIV-related stigma prevalence used in this analysis was limited to identifying higher levels of stigma above the mean score relative to lower levels below the mean, which may not allow for a suitable comparison with other measures of stigma. There may also be other unobserved and/or unmeasured factors that are associated with external HIV-related stigma that were not accounted for in the analysis. The modest but statistically significant findings reflect the strength of the observed relationship and represent a weak or small association. Nevertheless, this study provides insight into factors associated with the prevalence of HIV-related stigma and, therefore, contributes to the body of literature on this subject.

## Conclusion

The findings revealed that the prevalence of higher external HIV-related stigma varied by selected socio-demographic and health-related factors. The findings suggest a need for peer-facilitated HIV stigma reduction intervention targeting all types of educational institutions, including schools, and a need to strengthen work-based interventions to provide a supportive environment for PLHIV. The findings also highlight the importance of prioritising rural areas and accounting for the sociocultural context when developing and implementing HIV stigma reduction interventions in rural settings. Based on the current findings, efforts to mitigate externalised stigma should involve addressing public misconceptions about HIV, and stigma reduction should be considered a vital component of HIV testing and awareness.

## Supporting information

S1 Data Data review URL. http://dx.doi.org/doi:10.14749/1585345902.
(DOCX)

## References

[1] UNAIDS. The Gap report [Internet]. 2014. Available from: http://www.unaids.org/en/resources/campaigns/2014/2014gapreport/gapreport [cited 2020 Mar 11].

[2] Feyasa MB, Gebre MN, Dadi TK. Levels of HIV/AIDS stigma and associated factors among sexually active Ethiopians: analysis of 2016 Ethiopian Demographic and Health Survey Data. BMC Public Health. 2022;22:1080. doi: 10.1186/s12889-022-13505-1 35641915 PMC9158254

[3] Letamo G, Letamo R. Prevalence of, and factors associated with HIV/AIDS-related stigma and discrimination attitudes in Botswana. J Health Popul Nutr. 2020;39(1):347–357. 15038590

[4] Egbe TO, Nge CA, Ngouekam H, Asonganyi E. Stigmatization among People Living with HIV / AIDS at the Kumba Health. J Int Assoc Provid AIDS Care. 2020;19:1–7. 10.1177/2325958219899305 PMC6947670 31908184

[5] Sarkar T, Karmakar N, Dasgupta A, Saha B. Stigmatization and discrimination towards people living with HIV/AIDS attending the antiretroviral clinic in a centre of excellence in HIV care in India. Int J Community Med Public Health. 2019;6(3):1241–1246.

[6] Zegeye B, Adjei NK, Ahinkorah BO, Ameyaw EK, Budu E, Seidu A, et al. Individual-, household-, and community-level factors associated with pregnant married women’s discriminatory attitude towards people living with HIV in sub-Saharan Africa: A multicountry cross-sectional study. Wiley Health Science Reports. 2021. doi: 10.1002/hsr2.430 34746443 PMC8549109

[7] Mehlomakulu V. An assessment of external HIV-related stigma in South Africa: implications for interventions [dissertation]. Cape Town: University of Cape Town; 2021. Available from: https://open.uct.ac.za/handle/11427/33801

[8] SANAC. The People Living With HIV Stigma Index: South Africa 2014 [Internet]. [cited 2018 Jan 4]. Available from: http://sanac.org.za/2015/12/01/the-people-living-with-hiv-stigma-index-south-africa-2014-summary-report/

[9] Chambers LA, Rueda S, Baker DN, Wilson MG, Deutsch R, Raeifar E, et al. Stigma, HIV and health: A qualitative synthesis. BMC Public Health. 2015;15(1):848. 26334626 10.1186/s12889-015-2197-0 PMC4557823

[10] Seth P, Kidder D, Pals S, Parent J, Mbatia R, Chesang K, et al. Psychosocial functioning and depressive symptoms among HIV-positive persons receiving care and treatment in Kenya, Namibia, and Tanzania. Prev Sci. 2014;15(3):318–328. doi: 10.1007/s11121-013-0420-8 23868419 PMC4780409

[11] Logie CH, Lacombe-Duncan A, Wang Y, Kaida A, Conway T, Webster K, et al. Pathways from HIV-related stigma to antiretroviral therapy measures in the HIV care cascade for women living with HIV in Canada. J Acquir Immune Defic Syndr. 2018;77(2):144–153. doi: 10.1097/QAI.0000000000001589 29135650 PMC5770113

[12] Worede JB, Mekonnen AG, Aynalem S, Amare NS. Risky sexual behavior among people living with HIV/AIDS in Andabet district, Ethiopia: Using a model of unsafe sexual behavior. Front Public Health. 2022;10:1039755. doi: 10.3389/fpubh.2022.1039755 36579063 PMC9790964

[13] Pulerwitz J, Michaelis A, Weiss E, Brown L, Mahendra V. Reducing HIV-related stigma: lessons learned from Horizons research and programs. Public Health Rep. 2010;125(2):272–281. doi: 10.1177/003335491012500218 20297756 PMC2821857

[14] Ajong AB, Njotang PN, Nghoniji NE, et al. Quantification and factors associated with HIV-related stigma among persons living with HIV/AIDS on antiretroviral therapy at the HIV-day care unit of the Bamenda Regional Hospital, North West Region of Cameroon. Global Health. 2018;14:56. doi: 10.1186/s12992-018-0374-5 29866206 PMC5987427

[15] Brown A. ’How did a white girl get AIDS?’ Shifting student perceptions on HIV-stigma and discrimination at a historically white South African university. South African Journal of Higher Education. 2016;30(4):94‒111.

[16] Yang H, Li X, Stanton B, Fang X, Lin D, Naar-King S. HIV-related knowledge, stigma, and willingness to disclose: A mediation analysis. AIDS Care. 2006;18(7):717–724. doi: 10.1080/09540120500303403 16971280 PMC1933389

[17] Nabunya P, Byansi W, Sensoy Bahar O, McKay M, Ssewamala FM, Damulira C. Factors Associated With HIV Disclosure and HIV-Related Stigma Among Adolescents Living With HIV in Southwestern Uganda. Front Psychiatry. 2020;11:772. doi: 10.3389/fpsyt.2020.00772 32848940 PMC7411995

[18] Vanable PA, Carey MP, Blair DC, Littlewood RA. Impact of HIV-related stigma on health behaviors and psychological adjustment among HIV-positive men and women. AIDS Behav. 2006 Sep;10(5):473–82. doi: 10.1007/s10461-006-9099-1 16604295 PMC2566551

[19] Mall S, Middelkoop K, Mark D, Wood R, Bekker LG. Changing patterns in HIV/AIDS stigma and uptake of voluntary counselling and testing services: the results of two consecutive community surveys conducted in the Western Cape, South Africa. AIDS Care. 2013;25(2):194–201. doi: 10.1080/09540121.2012.689810 22694602

[20] Sullivan MC, Rosen AO, Allen A, Benbella D, Camacho G, Cortopassi AC, et al. Falling Short of the First 90: HIV Stigma and HIV Testing Research in the 90–90–90 Era. AIDS Behav. 2020;24:357–362. 31907675 10.1007/s10461-019-02771-7

[21] Khan R, Garman EC, Sorsdahl K. Perspectives on Self-Disclosure of HIV Status among HIV-Infected Adolescents in Harare, Zimbabwe: A Qualitative Study. J Child Fam Stud. 2023;32: 3775–3785.

[22] Simbayi LC, Zuma K, Zungu N, Moyo S, Marinda E, Jooste S, et al. South African National HIV Prevalence, Incidence, Behaviour and Communication Survey, 2017. Cape Town, South Africa: HSRC Press; 2019.

[23] Cloete A, Mabaso M, Maseko G, Jooste S, Mthembu J, Simbayi L, et al. Study Report: The People Living with HIV Stigma Index 2.0 in Six Districts of South Africa 2020–2021. Cape Town: Human Sciences Research Council; 2022.

[24] Statistics South Africa. Mid-Year Population Estimates 2017. Pretoria, South Africa: Statistics South Africa; 2017.

[25] Fabrigar L, Wegener D, MacCallum RSE. Evaluating the use of exploratory factor analysis in psychological research. Psychological Methods. 1999;4(3):272–299.

[26] DeVellis RF. Scale Development: Theory and Applications (Applied Social Research Methods Series, Vol. 26). Newbury Park, CA: Sage Publications; 1991.

[27] Ritsher JB, Otilingam PG, Grajales M. Internalized stigma of mental illness: psychometric properties of a new measure. Psychiatry Res. 2003 Nov 1;121(1):31–49. doi: 10.1016/j.psychres.2003.08.008 14572622

[28] Barke A, Nyarko S, Klecha D. The stigma of mental illness in Southern Ghana: attitudes of the urban population and patients’ views. Soc Psychiatry Psychiatr Epidemiol. 2011;46(11):1191–202. doi: 10.1007/s00127-010-0290-3 20872212 PMC3192946

[29] Brohan E, Elgie R, Sartorius N, Thornicroft G. Group G-ES: Self-stigma, empowerment and perceived discrimination among people with schizophrenia in 14 European countries: the GAMIAN-Europe study. Schizophr Res. 2010;122(1–3):232–8. 20347271 10.1016/j.schres.2010.02.1065

[30] Bifftu BB, Dachew BA. Perceived stigma and associated factors among people with schizophrenia at Amanuel Mental Specialized Hospital, Addis Ababa, Ethiopia: a cross-sectional institution based study. Psychiatry J. 2014;2014:694565. doi: 10.1155/2014/694565 24967300 PMC4055492

[31] Datiko D.G., Jerene D. & Suarez P. Stigma matters in ending tuberculosis: Nationwide survey of stigma in Ethiopia. BMC Public Health 20, 190 (2020). 32028914 10.1186/s12889-019-7915-6 PMC7006204

[32] Lapham J, Martinson ML. The intersection of welfare stigma, state contexts and health among mothers receiving public assistance benefits. SSM Popul Health. 2022 May 9;18:101117. doi: 10.1016/j.ssmph.2022.101117 35620484 PMC9127679

[33] Teshale AB, Tesema GA. Discriminatory attitude towards people living with HIV/AIDS and its associated factors among adult population in 15 sub-Saharan African nations. PLoS ONE. 2022;17(2):e0261978. doi: 10.1371/journal.pone.0261978 35120129 PMC8815885

[34] Ncitakalo N, Mabaso M, Joska J, Simbayi L. Factors associated with external HIV-related stigma and psychological distress among people living with HIV in South Africa. SSM—Population Health. 2021;14. doi: 10.1016/j.ssmph.2021.100809 34027011 PMC8121694

[35] Chory A, Nyandiko W, Martin R, Aluoch J, Scanlon M, Ashimosi C, et al. HIV-Related Knowledge, Attitudes, Behaviors and Experiences of Kenyan Adolescents Living with HIV Revealed in WhatsApp Group Chats. J Int Assoc Provid AIDS Care. 2021;20:1–11. doi: 10.1177/2325958221999579 33657911 PMC7940722

[36] Nyasulu PS, Tshuma N, Sigwadhi LN, Nyasulu J, Ogunrombi M, Chimoyi L. Factors associated with high HIV-related stigma among commuter populations in Johannesburg, South Africa. SAHARA-J: Journal of Social Aspects of HIV/AIDS. 2021;18(1):149–155. 10.1080/17290376.2021.1989022 PMC8555515 34702146

[37] Antabe R, Sano Y, Atuoye KN, Baada JN. Determinants of HIV-related stigma and discrimination in Malawi: evidence from the demographic and health survey. Afr Geogr Rev. 2022;42:594–606.

[38] The Global Network of People Living with HIV. HIV Stigma and Discrimination in the World of Work: Findings from the People Living with HIV Stigma Index; 2018.

[39] Iqbal S, Maqsood S, Zafar A, et al. Determinants of overall knowledge of and attitudes towards HIV/AIDS transmission among ever-married women in Pakistan: evidence from the Demographic and Health Survey 2012–13. BMC Public Health. 2019;19(1):793. doi: 10.1186/s12889-019-7124-3 31226969 PMC6588857

[40] Arefaynie M, Damtie Y, Kefale B, Yalew M. Predictors of Discrimination Towards People Living with HIV/AIDS Among People Aged 15–49 Years in Ethiopia: A Multilevel Analysis. HIV/AIDS (Auckland, NZ). 2021;13:283–292. 10.2147/HIV.S299812 PMC7979683 33758550

[41] Akatukwasa C, Getahun M, El Ayadi AM, Namanya J, Maeri I, Itiakorit H, et al. Dimensions of HIV-related stigma in rural communities in Kenya and Uganda at the start of a large HIV ’test and treat’ trial. PLoS ONE. 2021;16(5):e0249462. doi: 10.1371/journal.pone.0249462 33999961 PMC8128261

[42] Vlassoff C, Weiss MG, Rao S, Ali F, Prentice T. HIV-related stigma in rural and tribal communities of Maharashtra, India. J Health Popul Nutr. 2012;30(4):394–403. doi: 10.3329/jhpn.v30i4.13291 23304905 PMC3763610

[43] Turan B, Budhwani H, Fazeli PL, Browning WR, Raper JL, Mugavero MJ, et al. How does stigma affect people living with HIV? The mediating roles of internalized and anticipated HIV stigma in the effects of perceived community stigma on health and psychosocial outcomes. AIDS Behav. 2017;21(1):283–291. doi: 10.1007/s10461-016-1451-5 27272742 PMC5143223

[44] Moussa AB, Delabre RM, Villes V, et al. Determinants and effects or consequences of internal HIV-related stigma among people living with HIV in Morocco. BMC Public Health. 2021;21:163. doi: 10.1186/s12889-021-10204-1 33468093 PMC7815182

