# Supplementary material for: Prevalence and factors associated with external HIV-related stigma in the South African population: Results from the 2017 population-based household survey
Source: PLoS One. 2024 Sep 3;19(9):e0309694. doi: 10.1371/journal.pone.0309694 (PMC11371201; doi:10.1371/journal.pone.0309694)
Supplement: S1 Data — http://dx.doi.org/doi:10.14749/1585345902. (DOCX) [file pone.0309694.s001.docx]

**Data Review URL**

Data used in this analysis are available from the Human Sciences Research Council’s public data repository (data set). South African National HIV Prevalence, HIV Incidence, Behaviour and Communication Survey 2017: Combined. Version 1.0.

(http://dx.doi.org/doi:10.14749/1585345902.)
